# Haptools: a toolkit for admixture and haplotype analysis

**DOI:** 10.1093/bioinformatics/btad104

**Published:** 2023-02-27

**Authors:** Arya R Massarat, Michael Lamkin, Ciara Reeve, Amy L Williams, Matteo D’Antonio, Melissa Gymrek

**Affiliations:** Bioinformatics and Systems Biology Graduate Program, Department of Bioengineering, University of California San Diego, La Jolla, CA 92093, USA; Department of Computer Science and Engineering, University of California San Diego, La Jolla, CA 92093, USA; Department of Bioengineering, University of California San Diego, La Jolla, CA 92093, USA; 23andMe, Inc., Sunnyvale, CA 94086, USA; Department of Biomedical Informatics, Department of Medicine, University of California San Diego, La Jolla, CA 92093, USA; Department of Computer Science and Engineering, University of California San Diego, La Jolla, CA 92093, USA; Department of Biomedical Informatics, Department of Medicine, University of California San Diego, La Jolla, CA 92093, USA; Department of Medicine, University of California San Diego, La Jolla, CA 92093, USA

## Abstract

**Summary:**

Leveraging local ancestry and haplotype information in genome-wide association studies and downstream analyses can improve the utility of genomics for individuals from diverse and recently admixed ancestries. However, most existing simulation, visualization and variant analysis frameworks are based on variant-level analysis and do not automatically handle these features. We present haptools, an open-source toolkit for performing local ancestry aware and haplotype-based analysis of complex traits. Haptools supports fast simulation of admixed genomes, visualization of admixture tracks, simulation of haplotype- and local ancestry-specific phenotype effects and a variety of file operations and statistics computed in a haplotype-aware manner.

**Availability and implementation:**

Haptools is freely available at https://github.com/cast-genomics/haptools.

**Documentation:**

Detailed documentation is available at https://haptools.readthedocs.io.

**Supplementary information:**

Supplementary data are available at *Bioinformatics* online.

## 1 Introduction

Existing frameworks for complex trait analysis are typically based on variant-level analysis. However, phenotypic effects may also be mediated by haplotypes (Corder *et al.*, 1993; Williams *et al.*, 2014) (combinations of variants on the same chromosome) or by the local ancestry background on which a variant falls (Atkinson *et al.*, 2021; Naslavsky *et al.*, 2022). Incorporating these effects may improve the utility of genomic information for diverse and recently admixed individuals, but current tools have limited support for including these features. Here, we present haptools, an open-source toolkit for facilitating local ancestry aware and haplotype-based analysis of complex traits. Haptools supports fast simulation of admixed genomes, visualization of admixture tracks, simulating haplotype- and local ancestry-specific phenotype effects and computing a variety of common file operations and statistics in a haplotype-aware manner. Overall, haptools provides a valuable set of utilities for developing and benchmarking methods for ancestry-aware analysis of complex traits.

## 2 Features and methods

Haptools consists of a suite of command-line utilities and a corresponding Python library for performing simulations and common file operations on haplotypes, local ancestry labels and individual variants (Supplementary Figs S1 and S2, Table 1). Haptools is compatible with standard file formats as inputs and outputs, including VCF, PLINK and the newer PGEN format which results in greatly improved computational performance (Supplementary Fig. S3). In the following sections, we summarize the current core functionality available in haptools.

**Table 1.** Summary of current haptools utilities

Command	Description
simgenotype	Simulate admixed genomes
karyogram	Generate chromosome paintings for admixed individuals
simphenotype	Simulate phenotypes for complex traits with variant-, haplotype- or local ancestry-specific effects
transform	Obtain a VCF of pseudo-genotypes from a set of haplotypes
ld	Compute linkage disequilibrium between haplotypes (or genotypes) and a specific target haplotype
index	Sort, compress and index .hap files

### 2.1 .hap file format

Haptools implements a custom file format (*.hap) for flexible representation of haplotype-level and other information. These files consist of a collection of haplotypes. Each haplotype is defined by a set of one or more variants and their alleles, and optionally a local ancestry label, that tend to be inherited together on an individual chromosome (Supplementary Fig. S2). Unlike previous haplotype representations, the format is compatible with tabix (Li, 2011) and can be easily sorted and queried at the variant or haplotype level. Details and additional motivation for the .hap format are given in the Supplementary Methods.

### 2.2 Haptools simgenotype

The simgenotype utility simulates random mating between individuals of ancestral populations under a user-specified population history model, which defines admixture proportions and the number of generations of admixture. It outputs haplotype breakpoints and genotypes of simulated admixed individuals in VCF or PGEN format. simgenotype is adapted from admix-simu (Williams, 2016) with minor modifications to improve run time (Supplementary Material). We benchmarked simgenotype against admix-simu and AdmixSim2 (Zhang *et al.*, 2021) (Supplementary Fig. S4). While AdmixSim2 simulation run time is fastest, both AdmixSim2 and admix-simu require more run time overall because genotypes must be preprocessed into a custom input format. By contrast, simgenotype does not require additional preprocessing and supports directly simulating from file formats (VCF and PGEN) supported by large existing datasets such as the 1000 Genomes Project (Auton *et al.*, 2015).

### 2.3 Haptools karyogram

**Fig. 1.**
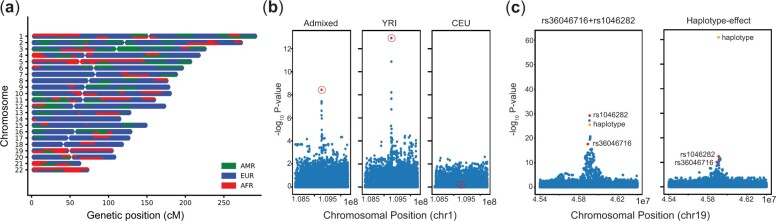
Example analyses performed using haptools. (**a**) An example karyogram depicting local ancestry tracts simulated by the simgenotype command. (**b**) Manhattan plot showing association summary statistics (−log10 *P*-values) for a trait with a single SNP (circled) simulated to be causal only when it occurs on an African haplotype. The SNP (rs12740374) is highly significant in simulated African but not European individuals. It has an intermediate *P*-value in a sample of simulated admixed individuals. (**c**) Manhattan plot showing association summary statistics for a trait simulated with either two causal SNPs (rs36046716 and rs1046282; left) or a single causal haplotype (composed of alleles from the two SNPs; right). Red = SNP-level *P*-values and orange = haplotype-level *P*-values for the variants of interest. When the haplotype is causal (right), it has a more significant *P*-value than the SNPs it is composed of. This large effect could be missed by variant-level association tests. Detailed methods underlying results shown in the figures are in the Supplementary Material.

karyogram takes breakpoints generated by simgenotype as input and generates a karyogram to visualize chromosome segments. It is adapted from an existing script (Martin, 2017). Example karyograms for individuals simulated under demographic models for admixed populations in the Americas are shown in Figure 1a and Supplementary Figure S5.

### 2.4 Haptools transform

transform produces a VCF file of pseudo-genotypes, in which each haplotype is encoded as a bi-allelic variant record, for a set of haplotypes in .hap format. This operation can facilitate downstream tasks which require variant-level information as input. For example, to perform association testing based on haplotypes, one could first use transform to generate a VCF encoding haplotypes as bi-allelic variants, and then use a standard framework such as PLINK (Chang *et al.*, 2015) to perform association tests.

### 2.5 Haptools simphenotype

The simphenotype utility simulates phenotypes for complex traits with variant-, haplotype- or local ancestry-specific effects. Causal haplotypes are loaded from a VCF file output by transform and their effect sizes are specified in a .hap file. By default, simphenotype simulates quantitative traits. Users may specify case–control traits by providing disease prevalence, which results in that percentage of individuals with the highest trait value being labeled as cases. We evaluated simphenotype by simulating quantitative traits under various scenarios, including individual causal variants (Supplementary Fig. S6), local ancestry effects (Fig. 1b, Supplementary Fig. S7), and haplotype-level effects (Fig. 1c).

## 3 Discussion

Accounting for ancestry and other more complex effects is becoming increasingly critical in association testing and downstream analysis pipelines. Haptools helps fulfill an unmet need to handle ancestry and haplotype level information in a standardized way that is compatible with existing file formats and workflows and is computationally efficient. It enables easily performing tasks such as genotype and phenotype simulation, local ancestry visualization and power analyses which previously have been done primarily using a variety of custom scripts. Overall, haptools will help enable more systematic incorporation of ancestry and haplotype-level features in future workflows.

## Data availability

The datasets used for validation and example generation are available from the haptools documentation page: https://haptools.readthedocs.io/en/stable/project_info/example_files.html, and the haptools-paper repository, https://github.com/CAST-genomics/haptools-paper.

## Supplementary Material

btad104_Supplementary_Data
