# Supplementary material for: Haptools: a toolkit for admixture and haplotype analysis
Source: Bioinformatics. 2023 Feb 27;39(3):btad104. doi: 10.1093/bioinformatics/btad104 (PMC9991497; doi:10.1093/bioinformatics/btad104)
Supplement: btad104_Supplementary_Data [file btad104_supplementary_data.pdf]

Supplementary Material - Haptools

Supplementary Note

The .hap file format

We present a new file format (.hap), designed to enable flexible representation of variant-, haplotype-, and local ancestry-level information, as well as to be extensible and compatible with existing tools. The format is human-readable but easy and quick to parse via command-line tools, ad-hoc scripts, and performant code. At the time of writing, no such format existed. A description and example of the .hap file format is shown in **Supplementary Fig. 2**.

Each haplotype in a .hap file is composed of a set of alleles that are inherited together on an individual chromosome. A .hap file is a tab delimited file containing different types of lines describing these haplotypes, each beginning with a symbol denoting the line type. Haplotype lines are prefixed by an H. These lines contain the haplotype's genomic coordinates as well its unique identifier. Variant lines are prefixed by a V and describe the alleles within a haplotype. These lines contain the ID of the haplotype to which they belong, the genomic coordinates of the variant, a variant ID, and the target allele. The genomic coordinate fields allow the file to be tabix indexed and subsequently queried to obtain either the haplotypes within a genomic region or the variant alleles within a haplotype. All commands within haptools take advantage of the indexing whenever it is available.

In addition to the required fields, extra fields are permitted at the end of each H and V line as long as they are declared and described in the header of the file. Different tools may require certain extra fields. For example, the transform command requires ancestral population labels for each haplotype when the --ancestry flag is used, while the simphenotype command requires an effect size for each haplotype. The ld command outputs linkage disequilibrium estimates in an extra field of the .hap file it outputs.

Compared to other formats for haplotypes, ours is more flexible but also easier to parse and faster to query. For example, PLINK1.9 (Purcell *et al.*, 2007) outputs a .blocks or .blocks.det file similar to our .hap file except that it does not store the alleles of each SNP in a haplotype. Instead, it encodes a list with each allele's variant ID. This design makes it impossible to specify haplotypes composed from combinations of REF and ALT alleles of different SNPs. By contrast, haplotypes in a .hap file are declared on separate lines from the alleles that they are composed of. The additional benefits of this strategy are that 1) extra, optional information can be appended to each allele, and 2) the alleles within a haplotype can be queried quickly via tabix in the same way that we query for haplotypes within a chromosomal region.

Notably, .hap files are not designed to store any kind of per-sample information. Instead, the transform command can be used to output haplotype-level "pseudo-genotypes" **(Supplementary Fig. 2)** for individual samples in VCF format, where it can optionally be easily transformed to more efficient formats such as BCF or PGEN by existing tools. By contrast, .hap files are optimized for storing metadata about haplotypes and their alleles. Thus, our intent is for the .hap file format to play a supporting role to VCF.

Supplementary Methods

An example ancestry-based phenotype association

In **Fig. 1b**, we used haptools to simulate an ancestry-based effect for the SNP with ID rs12740374. First, we simulated 10,000 admixed individuals resulting from admixture over 10 generations between a starting population of 65% CEU and 35% YRI individuals from the 1000 Genomes Project (Auton *et al.*, 2015) (hg38, lifted over from hg19). We also generated separate CEU and YRI populations of the same sample sizes without admixture. In each of the three simulated cohorts, we simulated a trait where the "T" allele of SNP rs12740374 was causal ($\beta = 0.08$) when it occurred on a haplotype originating from a YRI individual.

We chose this SNP because it has a similar MAF in CEU and YRI. Finally, we performed association testing with the simulated genotypes and phenotypes using PLINK2 (Chang *et al.*, 2015) with default parameters. The pipeline reproducing **Fig. 1b** is available at `https://github.com/cast-genomics/haptools-paper`.

Power to detect ancestry-dependent causal variants

In **Supplementary Fig. 7**, we analyzed the power to detect a variant that we designed to be causal in ancestry-dependent ways. We used the same populations and the same variant (rs12740374) as in **Fig. 1b**. This time, we simulated traits under two settings. In the first setting, the alternate allele of the SNP is causal only when it occurs on haplotypes descended from the original YRI population. In the second setting, denoted in orange as "normal", the alternate allele is causal regardless of the local ancestry background. We used simphenotype to simulate values for 100 traits under each of these settings and computed power as the proportion in which the causal SNP was significant ($p <$ 5e-8) in variant-level association tests performed using PLINK2 (Chang *et al.*, 2015) with standard parameters. The pipeline reproducing **Supplementary Fig. 7** is available at `https://github.com/cast-genomics/haptools-paper`.

Comparing SNP-based vs. haplotype-based association testing

In **Fig. 1c**, we used haptools to simulate traits from two SNPs (rs36046716 and rs1046282) in individuals from the 1000 Genomes Project (n=2,504, hg19) (Auton *et al.*, 2015). First, we used simphenotype to simulate a trait where both SNPs are independently causal (i.e. $y = \beta_1 x_1 + \beta_2 x_2 + \epsilon$). Next, we used transform and simphenotype to simulate a trait from a causal haplotype (h_1) composed from the "G" alleles of both SNPs (in this case $y = \beta_1 h_1 + \epsilon$) with $\beta = 0.2$ and $h^2 = 0.1$. Finally, we performed association testing for each phenotype using PLINK2 (Chang *et al.*, 2015) with standard parameters. The pipeline reproducing this figure is available at `https://github.com/cast-genomics/haptools-paper`.

Simgenotype implementation and benchmarking

simgenotype is based on admix-simu (Williams, 2016) and uses the same model file format to specify the admixture scenario, in which users may provide ancestry proportions in each generation, the number of generations of admixture, and the number of samples to output. The simulations include three or more ancestries: two or more source groups and one admixing population formed after the first generation and contributing to each subsequent generation. Simulation of admixed genomes is performed in two steps. In the first step, forward simulations of admixture are performed to generate haplotype breakpoints and the population origin of the adjacent segment. This process samples from pools of haplotypes of each ancestry at each recombination breakpoint according to the ancestry proportions in each generation. (Like many simulators, this approach does not fully account for long-range correlations in source haplotypes (Nelson *et al.*, 2020). In the second step, the resulting admixed genomes are output to a file. These steps are described below.

Step 1: Breakpoint simulation: Breakpoint simulation is performed one generation at a time. At each generation, we keep track of a number of haploid genomes equal to either 10,000 or ten times the number of samples specified in the model file (whichever is greater) in order to have a sufficient number of genomes to generate the final output set of individuals. Keeping track of a large number of haplotypes also reduces genetic drift by increasing the chance that the simulated samples' breakpoints are independent of each other.

At the first generation, or any subsequent generation when non-admixed population fractions are given, we randomly choose a population for each individual based on the population fractions specified in the model file and identify two parent genomes from that population. If the randomly chosen population for the individual is admixed, we randomly select two genomes generated from the past generation and label them as the parents.

Once parent genomes are chosen, individuals undergo a round of recombination where the markers within the input map file are used to simulate a recombination event between the parent chromosomes with probability $p(recombination) = 1 - e^{-m}$ where m is the distance in Morgans between two subsequent markers, based on a user-specified genetic map. The recombination process is applied independently to each chromosome. This newly generated haploid genome is stored and used in the next step of the simulation as either the previous generation or as the resulting admixed genomes in the final generation.

Step 2: Outputting genotypes: After breakpoints are generated, simgenotype outputs resulting admixed genomes to a VCF or PGEN file. This step is optional, and if it is not performed only the breakpoints are output. For each individual simulated, we randomly select two haploid genomes out of the population pool from the final generation of the simulation process. These genomes contain positional information about the span of the segments as well as the local ancestry of each segment. In order to convert this positional information into variants, we require a reference VCF or PGEN file and a mapping file that maps all samples within the reference VCF or PGEN file into populations. Each simulated genome includes all variants within the VCF or PGEN file. For each simulated segment, we copy the alleles that the segment spans from a randomly chosen haplotype from a reference individual with the same ancestry label as the segment.

Comparison to admix-simu: Our algorithm differs slightly from admix-simu's with an additional optimization utilizing a binary search instead of linear search when finding the starting point of the parent's segments to copy to the child after a recombination event. simgenotype also differs from admix-simu in the input and output file formats. admix-simu takes input and outputs in EIGENSTRAT genotype file format, whereas simgenotype takes input and output in VCF file format which also allows us to output additional format fields such as population labels for each allele in a simulated individual. simgenotype also supports input and output in PGEN format, which greatly improves run time of reading and writing of variant files, although does not accommodate custom format fields containing local ancestry metadata that are output in VCF mode. Finally, simgenotype does not require that the reference VCF have more samples in each population than are being output, since it samples haplotypes with replacement. Notably, this can result in bias if the number of ancestral haplotypes is small compared to the desired output sample size.

Notably, simgenotype, like admix-simu, does not consider more complex scenarios such as selection or variation in population size across generations.

Benchmarking simgenotype: We benchmarked simgenotype against admix-simu, as well as AdmixSim2 (Zhang *et al.*, 2021), a newer simulation tool that supports more complex simulations including incorporation of natural selection and mutation. We compared the run time of each major algorithmic step and distributions of the total number of breakpoints in each sample across the different tools. All experiments were tested in a Linux environment running Centos 7.4.1708 on a server with specifications of 125GB of RAM and 28 cores (Intel® Xeon® CPU E5-2660 v4 @ 2.00 GHz) and were performed on a single core.

For all runs, we subsetted to the first 30,000 variants on chromosome 20 in the 1000Genomes Phase 3 VCF and selected 100 samples each from the CEU and YRI populations. We then converted the subsetted VCF file to the different input genotype files required by admix-simu (EIGENSTRAT format) and AdmixSim2 (custom haplotype format). Overall, this conversion time for both the admix-simu and AdmixSim2 formats took around 10 minutes of compute time for chromosome 20.

For the admixture model, we simulated 100 individuals with a 50/50 mixture between YRI and CEU and varied the number of generations of admixture from 1 to 10. AdmixSim2 completed breakpoint simulation the fastest (range 0.05-1.33 seconds for 1-10 generations), followed by haptools simgenotype (4.51-41.57 seconds) and admix-simu (12-125 seconds). AdmixSim2 also wrote output files the fastest (0.10 seconds compared to 1.68 seconds for haptools simgenotype PGEN output and 5 seconds for admix-simu). Notably, for both AdmixSim2 and admix-simu, converting input VCF files to the correct format

took substantially greater compute time than the actual simulations (approximately 10 minutes), whereas haptools's simgenotype could process VCF files directly.

Finally, to assess the total number of breakpoints, we simulated 500 admixed individuals for between 1 and 6 generations of admixture, with 50/50 ancestry from CEU and YRI. We used the .bp files output by simgenotype and admix-simu to determine the distribution of the number of breakpoints in each sample, which resulted in nearly identical distributions across the two tools (**Supplementary Fig. 4**). Distributions are not shown for AdmixSim2 results, as the tool only outputs breakpoints when segments change populations, whereas the other tools generate breakpoints whenever a recombination event occurs, even if it occurs between two copies of a chromosome with the same population label. Thus the breakpoint results are not directly comparable. The expected distribution follows a $Poisson(\lambda)$ with $\lambda = r * d + c$ where r is the number of recombination events per meiosis which has been empirically derived to be 35.3 (McVean *et al.*, 2004), d is the total number of generations of admixture, and c is the number of chromosomes considered. Observed distributions match this expectation closely (**Supplementary Fig. 4**).

Demographic models for admixed American populations

Model files for **Fig. 1a** and **Supplementary Fig. 5** can be found in the haptools documentation (at https://haptools.readthedocs.io/en/stable/formats/models.html#more-example-files) and are based on a published reconstruction of the genetic history of the Caribbean (Moreno-Estrada *et al.*, 2013). Models are curated from Figure 3 of that study, which gives a approximate timelines (measured in number of generations) of major admixture events, driven by incoming migrants from Europe or Africa to various regions of the Caribbean.

Commands used for generating karyograms

Code used to generate **Fig. 1a** and **Supplementary Fig. 5** is provided below. **Fig. 1a**. is the Puerto Rico simulation of the demographic simulations found in **Supplementary Fig. 5**. The *./maps/* directory can be downloaded from Beagle: `https://haptools.readthedocs.io/en/stable/formats/maps.html`

```
Figure 1a. and Supplementary Figure 5.
for model in puertorico honduras colombia dominica
do
    haptools simgenotype \
        --model haptools/tests/data/dat_files/${model}.dat \
        --mapdir ./maps/ \
        --chroms 1,2,3,4,5,6,7,8,9,10,11,12,13,14,15,16,17,18,19,20,21,22 \
        --ref_vcf NA \
        --sample_info NA \
        --seed 1 \
        --out ./${model}_results \
        --only_breakpoint

    haptools karyogram \
        --bp ./${model}_results.bp \
        --sample Sample_1 \
        --out ./${model}_results.pdf \
        --title "${model}" \
        --centromeres haptools/tests/data/centromeres_noX_hg19.txt \
```

```
        --colors EUR:blue,AFR:red,AMR:green
done
```

Supplementary Figures

Supplementary Figure 1

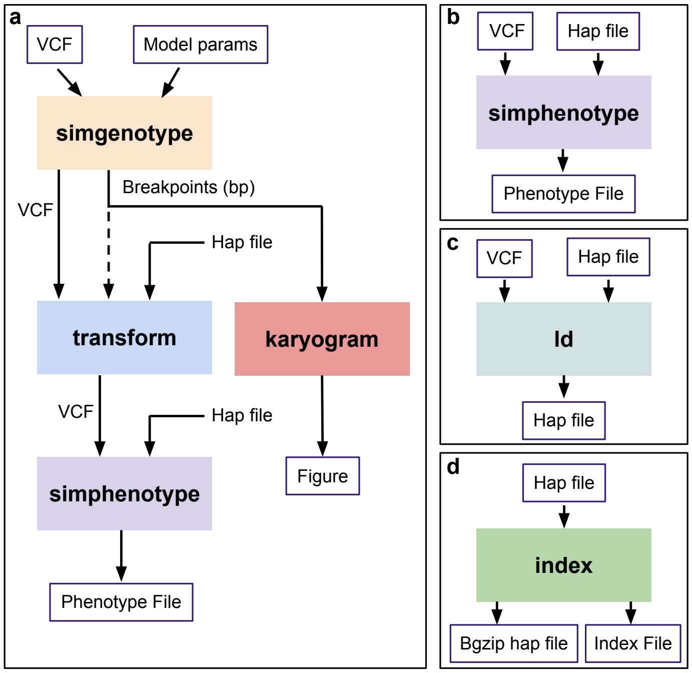

Figure 1: Overview of haptools utilities. Schematics of the six main haptools subcommands (simgenotype, transform, karyogram, simphenotype, ld, and index), and their typical usage pipelines. **(a)** Main simulation pipeline with the goal of simulating a phenotype derived from ancestry-specific effects in admixed individuals. simgenotype takes in a reference VCF file and model parameters and outputs a VCF and breakpoints file for simulated admixed individuals. The breakpoints file can then be used in the karyogram subcommand to generate a karyogram visualizing all the ancestry tracts in each chromosome for a particular sample. Alternatively, the VCF output by simgenotype can then be passed into the transform subcommand along with a haplotype file in order to obtain a VCF file containing haplotype-level information. Lastly, the VCF output from transform along with the haplotype file can be passed into simphenotype resulting in a phenotype file in the .pheno file format which can be used for downstream association analyses or other pipelines. **(b)** simphenotype takes in a standard VCF file and haplotype file and simulates a complex trait taking into account haplotype or local ancestry specific effects. **(c)** The ld subcommand takes in a VCF file and haplotype file and computes the pairwise linkage disequilibrium between a single target haplotype and each variant or haplotype in the VCF file. It outputs a new haplotype file containing the computed LD values in an extra field. **(d)** The index subcommand sorts, bgzips, and tabix-indexes a target hap file to enable fast querying by genomic location or haplotype ID.

Supplementary Figure 2

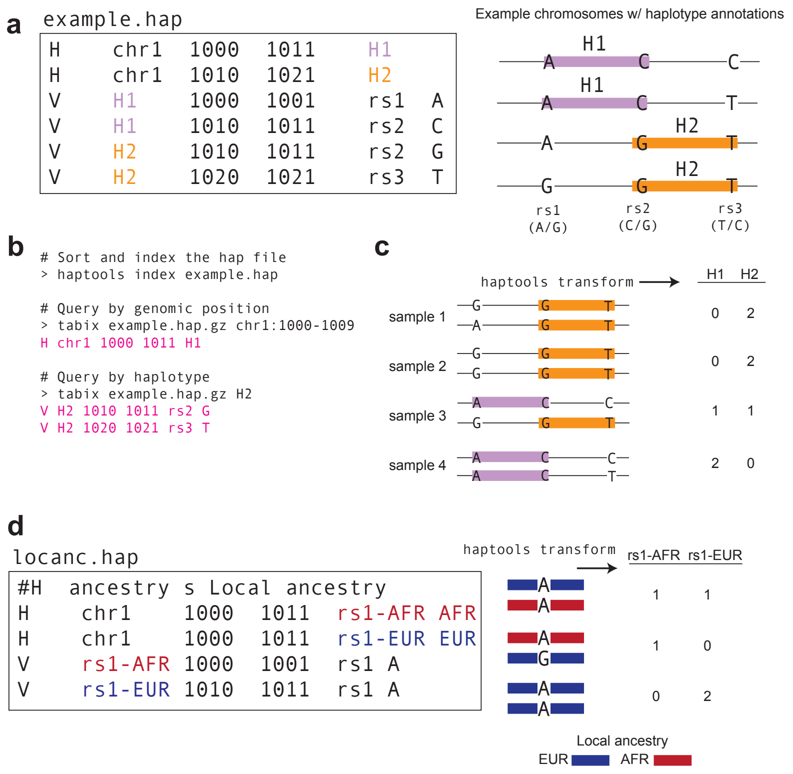

Figure 2: Overview of the .hap file format. **(a)** Example .hap file. Lines beginning with an "H" denote haplotypes. Each haplotype is described by its genomic position (columns 2-4), unique ID (column 5), and optional additional fields. Lines beginning with a "V" denote variants within a haplotype. Variants are described by the haplotype ID on which they fall (column 2), chromosome coordinates (columns 3-4), the variant ID (column 5), and allele (column 6). Example chromosomes are annotated with their matching haplotypes on the right. **(b)** Example commands to index and query .hap files. Once indexed, .hap files can be queried by genomic position or haplotype ID using tabix. **(c)** Example output of haptools transform. Four example diploid samples are shown. transform outputs the number of copies of each haplotype of interest in each sample. The result is a new VCF file in which haplotypes can be treated similarly to individual variants in downstream analyses such as GWAS. **(d)** Example .hap file denoting local ancestry labels. Ancestry may be included as an optional field which must be defined in the header of the .hap file. In this case, the sixth column of the "H" lines denotes the local ancestry label. The results of haptools transform only count haplotype copies that match the variants of that haplotype as well as the specified local ancestry label. This enables performing downstream analysis such as GWAS in a local-ancestry aware manner to assign specific effect sizes to variants that depend on the local ancestry background on which they fall.

Supplementary Figure 3

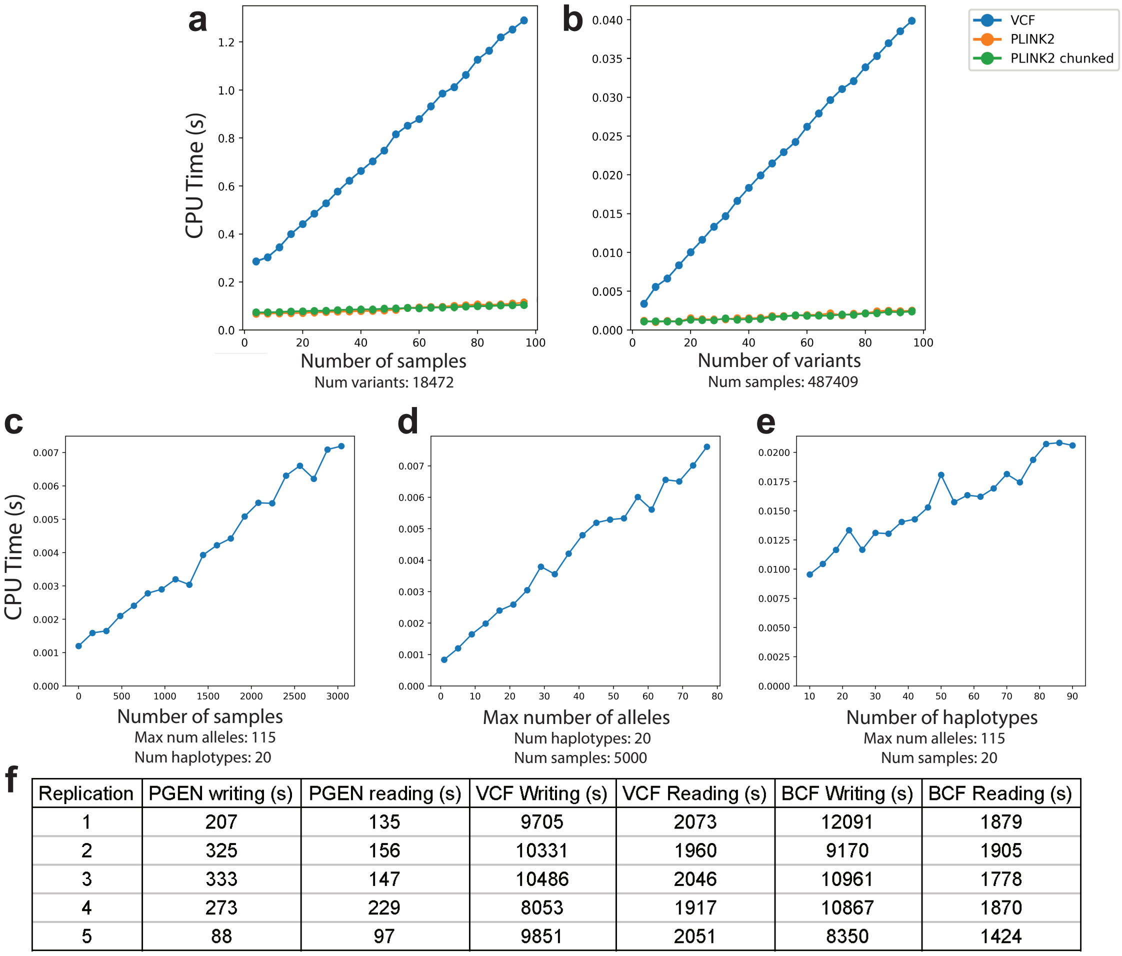

f

Replication	PGEN writing (s)	PGEN reading (s)	VCF Writing (s)	VCF Reading (s)	BCF Writing (s)	BCF Reading (s)
1	207	135	9705	2073	12091	1879
2	325	156	10331	1960	9170	1905
3	333	147	10486	2046	10961	1778
4	273	229	8053	1917	10867	1870
5	88	97	9851	2051	8350	1424

Figure 3: Benchmarking haptools run time. **(a)** and **(b)** compare computational efficiency of loading variants from VCF vs PGEN files. We measured the time to load: **(a)** 18,472 variants and a variable number of samples, and **(b)** 487,409 samples and a variable number of variants. Each file was composed from SNPs in the UK Biobank Project (Liang *et al.*, 2021). Time scales linearly for both VCF files (blue) and PGEN files (orange) as a function of both variables, but run time is greatly reduced for PGEN files. This does not change when the PGEN file is read in chunks (green) to reduce overall memory usage. Panels **(c-d)** analyze the time required to transform haplotypes. We first selected genotypes and haplotypes from the UK Biobank Project (Liang *et al.*, 2021). Then, we measured the time required to transform haplotypes while varying **(c)** the number of samples, **(d)** the maximum number of alleles in a haplotype, and **(e)** the number of haplotypes. The time scales linearly as a factor of all three variables. **(f)** Seconds required to read and write 100,000 samples and 30,000 variants from PGEN, VCF, and BCF files.

Supplementary Figure 4

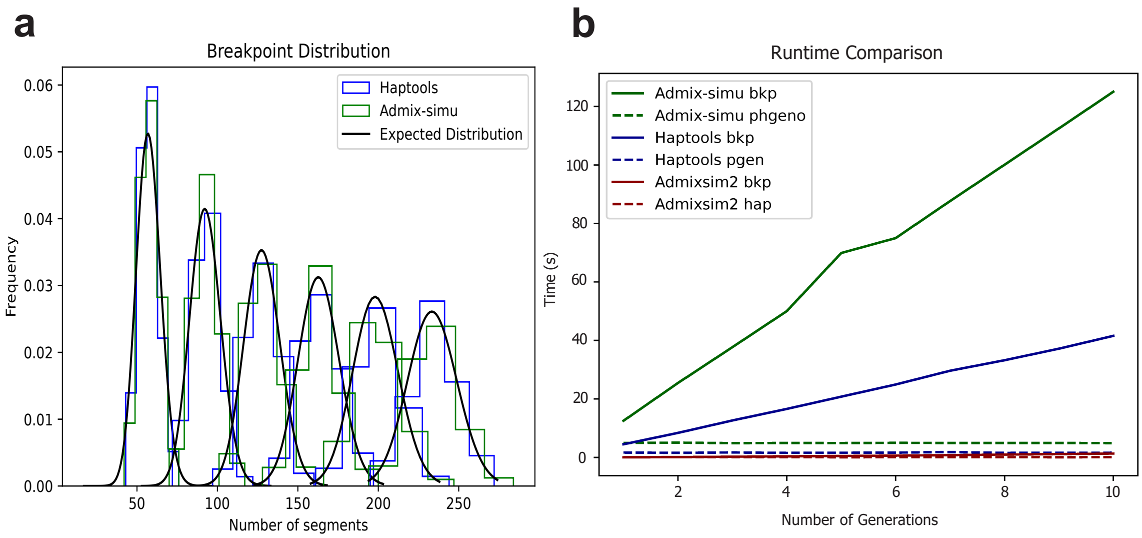

Figure 4: Comparing haptools simgenotype to other admixture simulation methods. **(a)** Comparison of the distributions of the total number of breakpoints in 500 simulated genomes. The leftmost distribution is for one generation of admixture, and the rightmost is for six generations of admixture. Blue=haptools simgenotype, green=admix-simu, black=expected distribution. Breakpoint distributions are not shown for AdmixSim2 as they were found to not be directly comparable (see Supplementary Methods). **(b)** Run time comparison between haptools simgenotype, admix-simu, and AdmixSim2. The run time is broken up into two main components: simulation run time (bkp), and genotype output run time (phgeno, pgen, and hap).

Supplementary Figure 5

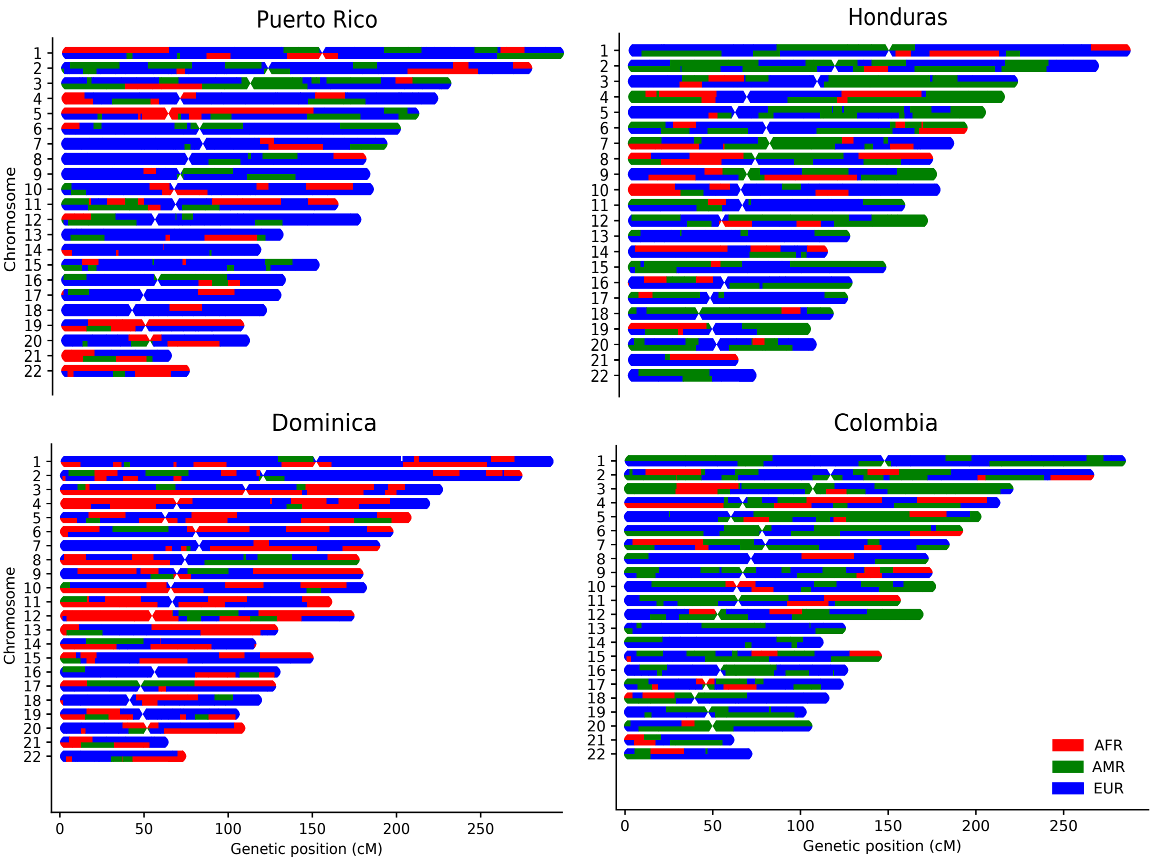

Figure 5: Karyograms for individuals simulated under Caribbean admixture models. Results of simulating Caribbean populations based on analysis of their genetic history and inferred migration origins (Moreno-Estrada *et al.*, 2013) (see Supplementary Methods). These populations had three main sources introduced throughout their history: African (AFR), European (EUR), and Native American (AMR) and the analysis dated back to a range of 14-17 generations ago.

Supplementary Figure 6

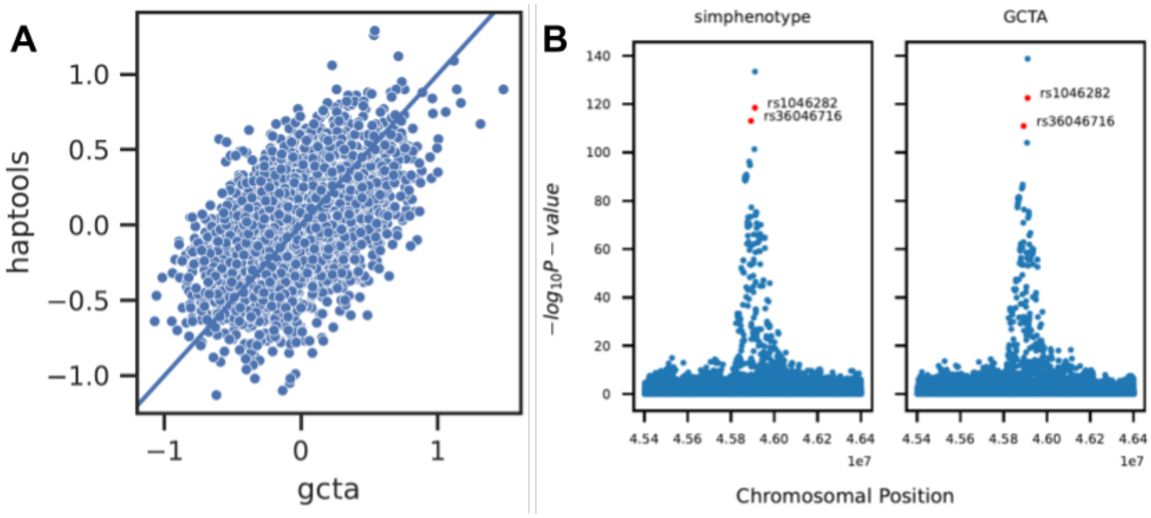

Figure 6: Standardized phenotypes produced by haptools simphenotype and GCTA (Yang *et al.*, 2011). Two SNPs (rs36046716 and rs1046282) from the 1000 Genomes Project (Auton *et al.*, 2015) (hg19, n=2504) were used as independent, causal variables with effect sizes of 0.2 and a heritability of 0.5. **(a)** shows a comparison of simulated phenotypes from each tool for each sample. **(b)** shows Manhattan plots with association summary statistics for a trait simulated with haptools simphenotype (left) and GCTA (right).

Supplementary Figure 7

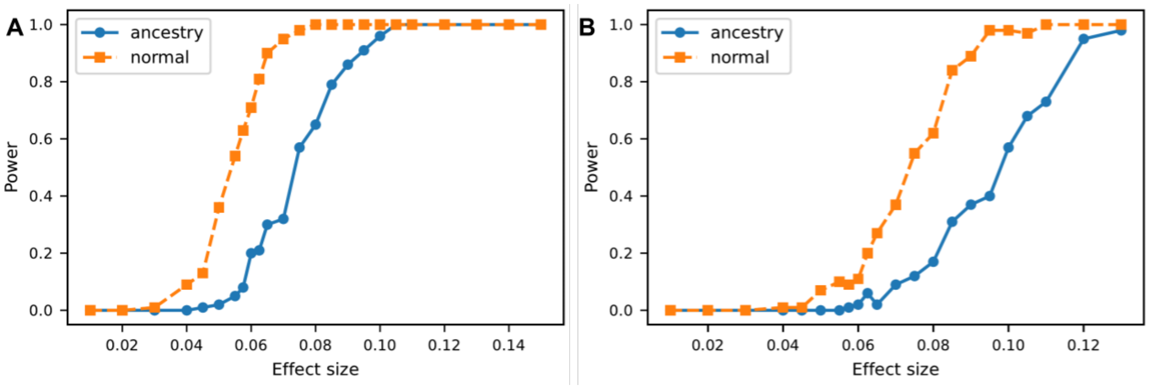

Figure 7: Power to detect the causal variant in simulated **(a)** quantitative and **(b)** case/control traits. We used haptools simgenotype to create an admixed population from YRI and CEU individuals in the 1000 Genomes Project (Auton *et al.*, 2015) in a 1 Mbp region around a particular SNP. The SNP (rs12740374) was chosen because it has a similar MAF in individuals of European and African ancestry. Then, we used haptools transform and haptools simphenotype to perform phenotype simulation under two settings. In the first setting, denoted in blue as “ancestry”, the ALT allele of the SNP is causal only when it occurs on haplotypes descended from the original YRI population. In the second setting, denoted in orange as “normal”, the ALT allele is causal regardless of the ancestral background. Power is defined as the proportion of simulations (out of a total of 100 replications) in which the causal SNP was significant ($p \leq$ 5e-8) in a standard variant-level association test with the simulated genotypes and phenotypes. As expected, power to detect an effect using standard association testing is lower when the effect is conditioned on the ancestral background.

References

Auton, A. *et al.* (2015). A global reference for human genetic variation. *Nature*, **526**(7571), 68–74.

Chang, C. C., Chow, C. C., Tellier, L. C., Vattikuti, S., Purcell, S. M., and Lee, J. J. (2015). Second-generation PLINK: rising to the challenge of larger and richer datasets. *GigaScience*, **4**(1). s13742-015-0047-8.

Liang, J. *et al.* (2021). Educational attainment protects against type 2 diabetes independently of cognitive performance: a mendelian randomization study. *Acta Diabetologica*, **58**(5), 567–574.

McVean, G. A., Myers, S. R., Hunt, S., Deloukas, P., Bentley, D. R., and Donnelly, P. (2004). The fine-scale structure of recombination rate variation in the human genome. *Science*, **304**(5670), 581–584.

Moreno-Estrada, A. *et al.* (2013). Reconstructing the population genetic history of the Caribbean. *PLoS Genet*, **9**(11), e1003925.

Nelson, D., Kelleher, J., Ragsdale, A., Moreau, C., McVean, G., and Gravel, S. (2020). Accounting for long-range correlations in genome-wide simulations of large cohorts. *PLoS Genet*, **16**(5), e1008619.

Purcell, S. *et al.* (2007). PLINK: a tool set for whole-genome association and population-based linkage analyses. *Am J Hum Genet*, **81**(3), 559–575.

Williams, A. (2016). admix-simu: admix-simu: program to simulate admixture between multiple populations. *10.5281/zenodo.45517*.

Yang, J., Lee, S. H., Goddard, M. E., and Visscher, P. M. (2011). GCTA: a tool for genome-wide complex trait analysis. *Am J Hum Genet*, **88**(1), 76–82.

Zhang, R., Liu, C., Yuan, K., Ni, X., Pan, Y., and Xu, S. (2021). AdmixSim 2: a forward-time simulator for modeling complex population admixture. *BMC Bioinformatics*, **22**(1), 506.
